# Early Treatment of Acute Complex Regional Pain Syndrome after Fracture or Injury with Prednisone: Why Is There a Failure to Treat? A Case Series

**DOI:** 10.1155/2016/7019196

**Published:** 2016-04-30

**Authors:** Paul Winston

**Affiliations:** Department of Rehabilitation Medicine, University of British Columbia, Victoria, BC, Canada V8Z 6R5

## Abstract

*Background*. Complex regional pain syndrome (CRPS) after fracture is a cause of pain, dysfunction, and potentially permanent disability. The evidence for treatment with oral corticosteroids is growing and supported by several international guidelines; however, treatment is not widely offered.* Objective*. Rapid recognition and treatment of complex regional pain in the upper extremity after acute injury as a disease modifying and potentially curative treatment.* Methods*. The present study was a case series involving three patients who developed CRPS after a trauma to the neck and/or upper limb. Patients were screened by clinical examination and bone scan and met the Budapest criteria.* Results*. Resolution of pain, swelling, and disability in all three patients.* Discussion*. There is increasing support, based on the existing evidence and clinical outcomes, for the use of prednisone to treat the acute phase of CRPS and as a promising treatment to halt the progression of the phenomenon and potentially cure the condition; however, widespread use of prednisone likely remains low, potentially resulting in long-term pain, joint contracture, and disability. A large-scale randomized control trial has not been performed.* Conclusion*. Corticosteroids can be an effective treatment option for CRPS after fracture.

## 1. Introduction

In 1872, Silas Weir Mitchell pondered the etiology of the pain syndrome currently termed complex regional pain syndrome (CRPS). In the 21st century, CRPS remains a debated syndrome, and multiple definitions, criteria, and treatment options exist. Institutional preferences often determine the course of treatment, which may lead to delayed recognition and treatment of the modifiable early stages of the disease.

The incidence of CRPS varies widely. A population study in the Netherlands [1] suggested the incidence to be 26.2 cases per 100,000 person-years. While there are many etiologies, a 2003 review of Olmsted County and Mayo Clinic records reported that a fracture was the trigger for CRPS in 46% of cases [2]. Guidelines regarding diagnosis and treatment vary widely. Harden et al. [3] suggest that the Budapest criteria for CRPS are more sensitive and specific than the International Association for the Study of Pain criteria [4]. In their 2013 Cochrane review, O'Connell et al. [5] concluded that “there is currently no strong consensus regarding the optimal management of complex regional pain syndrome although a multitude of interventions have been described and are commonly used.” In their 2010 article in Pain, Fischer et al. [6] noted that a lack of positive studies could be due to the fact that “these treatments were applied in heterogeneous groups of CRPS-1 patients, without accounting for possible differences related to prevailing pathophysiological mechanisms in individual patients.”

Despite a lack of consensus, Kingery [7], in a 1997 critical review in Pain, claimed that there is level 1 evidence to support corticosteroids as a disease modifying treatment in the early phase. The 2013* CRPS: Practical Diagnostic and Treatment Guidelines, 4th edition* [4], sponsored by the Reflex Sympathetic Dystrophy Syndrome Association, also suggest that “oral corticosteroids are the only anti-inflammatory drug for which there is direct clinical trial evidence in CRPS.” The authors report this claim as level 1 evidence using the definition of either a meta-analysis or systemic review in this article [4]. Fischer et al. [6] further support that there is a high-quality randomized control trial that supports the use of oral prednisolone. Harden et al. [4] wrote that there is very little high-quality research but “nevertheless in this ‘evidence vacuum' we still have the responsibility to treat.” The lack of a large randomized control trial likely accounts for why patients frequently are not offered corticosteroid treatment after fracture or injury in a timely manner. Clinicians may not be aware of this option as a treatment. A Cochrane review of the subject does not support the level 1 claim [5].

Failure to treat early may result in lifelong pain, loss of function, or even amputation; unemployment and prolonged disability are common. Early recognition of CRPS, with acute inflammatory changes, may lead to rapid and effective treatment with corticosteroids. Early treatment can lead to near resolution of the syndrome and the prevention of long-term pain, loss of function, and disability [8, 9].

The most common cause of active acute CRPS is in an upper extremity after fracture [1, 2, 10]. However, the fracture does not need to be at the site of the future CRPS; as this case series will demonstrate, spinal cord injury and nerve root compromise can cause CRPS in an upper extremity. Estimates of incidence of CRPS after fracture vary widely, from 1% to 37% [10–12]. This large range likely reflects the challenge of using the existing diagnostic criteria clinically. The patient typically presents weeks to months after the initial event with a swollen painful hand and wrist [9, 10, 12, 13]. Function in the hand is lost as symptoms progress. In the upper extremity, the tell-tale signs are overt, with swelling of the entire wrist and hand. There is loss of normal “wrinkling” over the knuckles. Flexion of the digits occurs due to contracture of the interphalangeal joints. The loss of wrist range may be disabling. The patient is no longer able to tuck the finger tips in to the palm with fisting or extend at the metacarpophalangeal (MCP) joint or interphalangeal joints. Hypersensitivity and allodynia are typically seen. Pain during an MCP compression test can be a useful sign [14]. The expected temperature (sudomotor) and skin changes associated with CRPS are often seen [4]. Though not discussed in the present series, the condition is not limited to the arm and may be exhibited in a lower extremity.

The pathophysiology of CRPS remains a subject of extensive study. The role of corticosteroids in the treatment of CRPS is attributed to the proinflammatory state and autoimmune mechanisms that have been proposed in an increasing body of literature. Fischer et al. [6] offer a proposed pathway for an inflammatory cascade as a result of an exaggerated inflammatory response to tissue injury. Elevated cytokine and mast cell levels and other inflammatory markers have been described [6, 13, 15]. A systematic review involving 22 studies in 2012 [16] cited the presence of a proinflammatory state in the blood, blister fluid, and cerebral spinal fluid in CRPS. Autoimmune mechanisms point to antibody formation, proinflammatory monocytes, and B cells among the cascade [12, 16, 17].

The anti-inflammatory properties of corticosteroids have been described for over three decades. The often cited 1982 study by Christensen et al. [18] noted that all 13 steroid-treated patients reported 75% improvement at three weeks, while only two patients in the placebo group did. A randomized controlled trial by Kalita et al. [19] examined 60 patients with CRPS after a stroke; 83.3% showed improvement with prednisolone versus 16.7% in the control group treated with piroxicam. The improvement in shoulder-hand syndrome after stroke was also noted by a placebo-controlled study involving 36 patients in which 31 were improved within 10 days of treatment [20]. A 2006 study [8] noted significant pain reduction and improvements in functional abilities in the hand and joint range of motion in a study involving 31 patients with CRPS, 26 of which had CRPS due to fracture. Patients were treated with doses starting at 40 mg to 60 mg of prednisone and titrated over several weeks; improvements persisted at one year. The authors noted that treatment with corticosteroids could offer a pain-free condition [8]. In a 2014 retrospective study involving 45 patients with CRPS of an upper extremity after traumatic upper limb injury who were treated with 30 mg per day of prednisolone tapered over five weeks, the patients were found to have significant improvements in clinical symptoms [9]. Several other protocols for dosing have been used. These include tapering doses over weeks starting at 40 mg [19]. Other articles suggest a starting dose of 40 mg to 60 mg, tapering over several weeks [8, 21]. Bianchi et al. [8] noted that doses of 80 mg to 200 mg have been trialed.

## 2. Methods

Acute CRPS was assessed in patients with antecedent trauma or neurological injury with the pertinent findings of a painful, unilateral, hot, and swollen arm or hand and wrist, sudomotor changes, capsular thickening of joints, and loss of range of motion in multiple joints of the hand, wrist, or shoulder. Bruehl [13] noted that while it is not a formal category, acute CRPS refers to “warm CRPS,” associated with a warm, red, and edematous extremity.

The patients in the present case series met the Budapest criteria [3]. All patients fulfilled the following:

1. Continuing pain was disproportionate to the inciting event.
2. There were symptoms of motor or trophic changes and vasomotor, sensory, and sudomotor/vasomotor changes.
3. There were signs of motor or trophic changes and vasomotor, sensory, and sudomotor/vasomotor changes.
4. As the pain, swelling, and loss of range were noted in fingers, wrists, and shoulders beyond the injured structures, there was no other diagnosis that would better explain the condition.

In his 2015 British Medical Journal review, Bruehl [13] noted that CRPS remains a clinical diagnosis. The workup for the following cases relied on widely available tests to rule out other causes of a painful inflamed limb, including blood work that investigated other causes of inflammation. A complete blood count and C-reactive protein (CRP) levels were investigated to rule out infectious or inflammatory causes. A triple-phase bone scan is a widely available test that may show increased uptake at the small joints of the wrists and hands [21–23]. An X-ray may correlate with periarticular osteopenia within weeks of the onset of this condition. A swollen joint or a history of gout merits a workup for that condition. Lack of access to specialized sudomotor or sensory testing precluded their use. On diagnosis, a titrating dose of 60 mg of prednisone with a taper of 5 mg per day was administered until 20 mg was used. This dose was chosen because it has been classically described as a starting dose and was common among Canadian physiatrists [21]. Patients were seen weekly. The final 20 mg was weaned as symptoms subsided. Treatment lasted <1 month in all cases.

## 3. Case Presentation

**Figure 1:**
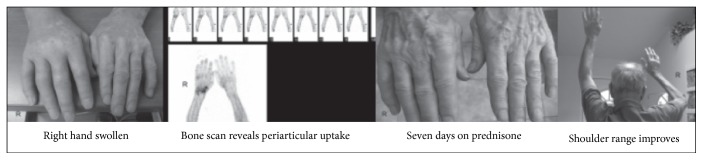
Case 1: a 78-year-old man with a central cord syndrome due to a flexion extension injury causing disc herniation and cord compression. After surgery, he regained the ability to walk with slow improvement of arm function. Four months later, he developed painful swelling of the right hand and loss of range in the denervated right shoulder, with loss of use of the right hand.

*Case 1*. A 78-year-old man experienced a fall and hyperextension injury to his neck. Traumatic disc herniation caused central cord syndrome, the predominance of the arms weaker than of the legs. He underwent anterior discectomy. Postoperatively, his arm weakness resolved well. He was able to walk independently and use his arms purposely at time of discharge from the rehabilitation unit. Four months after injury, he developed painful swelling of the right hand. He reported severe pain, swollen joints, and the inability to use the right hand. His neurosurgeon ordered urgent magnetic resonance imaging when notified of his symptoms, which was reported as unremarkable. A chance meeting between the patient and the present author at the hospital led to the recognition of the acutely inflamed hand. C-reactive protein level and white blood cell count were normal. An urgent triple-phase bone scan revealed diffuse hyperemia in the right hand and wrist, with the left arm being normal. Clinical examination revealed a grossly swollen hand. The MCP and interphalangeal joints were contracted. He was unable to tuck and fist his fingers or passively extend to neutral at any of these joints. His shoulder range was restricted with a capsular pattern on the right. There was hypersensitivity to touch at the hand, with temperature fluctuations of hot and cold. There was pain with compression of the joints, the greatest at the MCP joints. The decision to treat immediately with prednisone was made. The patient kept a journal of his findings. At one week, the hand had returned to its normal morphology with no pain. The shoulder range of motion also improved. He tapered his analgesia until it was fully weaned over a short time period. After review, the patient was weaned off the prednisone over the next two weeks. The patient's hand remained normal, and swelling and pain did not reoccur (see Figure 1).

**Figure 2:**
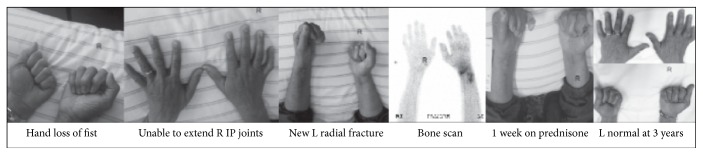
Case 2: a 50-year-old diabetic woman fell on ice fracturing her right wrist requiring open reduction internal fixation (ORIF). She developed capsular swelling in the right hand and lack of full flexion and extension at the interphalangeal joints; hand therapy was provided. When assessed 10 months after the accident, there was ongoing loss of range of motion. Shortly after, a motor vehicle accident resulted in a left radial fracture with an ORIF, with capsular restrictions in the wrist and hand, and loss of supination. A bone scan showed activity on the left only. Prednisone was initiated with little change on the old right injury but resolution of pain and loss of range on the new left injury.

*Case 2*. A 50-year-old non-insulin-treated woman with diabetes sustained a right distal radius fracture after a slip on ice. She was casted for six weeks and developed right hand and wrist pain by the end of the six weeks. X-rays revealed that the fracture healed well. She was assessed one year after injury and reported diffuse hand and wrist pain. She was unable to make a complete fist on the right, and there was loss of joint extension. In particular, the 5th digit was contracted at the interphalangeal joints; the hand was not uniformly swollen. The digits were all flexed at rest. Due to the late presentation and maturity of the contractures, she was sent for hand therapy, an extension splint, and a bone scan. Four days after this visit, she was involved in an accident and sustained a left wrist fracture of the distal radius and ulna. She required open reduction internal fixation and underwent a bone scan several weeks after the second fracture. The right side, the older fracture, showed no significant periarticular uptake. In contrast, on the newly fractured left hand, there was diffuse hyperemia in the left wrist, distal forearm, and fingers, as well as uptake at the left sided ribs in the 5th, 6th, 7th, and 10th ribs, consistent with fractures. On the newly fractured left arm, she had developed swelling in the digits and capsular restrictions. The 5th digit was difficult to extend. There was an obvious deformity in the left wrist. She experienced pain with MCP compression. While the right hand had continued to settle, the left hand was acutely swollen and a diagnosis of CRPS was made. She was treated with the titrating dose of prednisone with instructions to monitor her blood sugars. The inflammation rapidly improved; however, her blood sugars proved difficult to control and her weaning was escalated. She gained improvement in the left hand and wrist, with the return of normal range of motion, skin contour, and wrinkling of the phalangeal joints. On the original right hand, she remained unable to fully extend the digits, and a right 5th digit contracture remained (see Figure 2).

**Figure 3:**
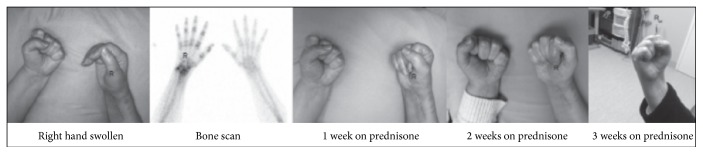
Case 3: a 50-year-old woman fell, sustaining an incomplete cervical spinal cord injury, right wrist fracture, and pulmonary emboli. She had excellent motor recovery but one month after discharge she developed a painful swollen right hand. When she was seen at two months, the pain had peaked but it was painful with metacarpophalangeal joint compression and she had very little function in the right hand due to an inability to fist the hand.

*Case 3*. A 50-year-old woman presented with traumatic incomplete quadriplegia due to a fall. A right wrist fracture, deep vein thrombosis of the leg, and pulmonary emboli were among numerous medical complications. Limb power returned slowly, and she was stabilized and discharged home. A follow-up had been arranged for two months later. After discharge, she developed a swollen painful right hand. There was loss of wrist supination and inability to form a fist. She experienced pain with compression of the interphalangeal joints; however, the patient reported that it was not as severe as in the weeks before the appointment. Sensation was altered, with temperature changes noted. Nerve conduction tests were performed of the median and ulnar nerves and were within normal limits. A bone scan showed extensive periarticular uptake in the wrist and small joints of the hand. A diagnosis of CRPS was made and treatment with prednisone was initiated. Rapid improvement was made over the next two weeks and continued after therapy was completed. Her hand function normalized (see Figure 3).

## 4. Discussion

The treatment of CRPS presents a significant challenge when the patient presents subacutely or late for treatment. There are no validated guidelines to direct care. Several of the research tests recommended are not widely available and could drastically prolong the treatment of CRPS, including thermal quantitative sensory testing, magnetic resonance imaging, and other sudomotor and sensory testing [3]. This points back to the evidence vacuum.

Regardless, as Harden et al. [4] argue, there must be treatment. His group additionally noted that the classic phases may not be observed. However, in the acute phase after a trauma, the presentation is consistent with the appearance of an acute inflammatory disorder, with recognizable morphological changes, pain, possible periarticular uptake on bone scan, pain with MCP compression, and lack of range of motion in multiple joints. The third case reveals that the pain may subside; however, painful capsules and contracture may progress until the hand is not usable.

Many treatments have been recommended and studied. After fracture, rapid recognition and treatment with prednisone may alter the course of this process and prevent significant disability [8, 9]. This readily available treatment shows efficacy within days of treatment and typically is offered for a short course of three to four weeks and is generally well tolerated. The use of corticosteroids has been investigated in sufficient studies, with leading authors in consensus papers supporting their use. As Bruehl [13] recently noted, they are often used by those familiar with the condition in patients to treat the early inflammatory component of the disease. The danger of not treating urgently is a painful, contracted limb with permanent rheological changes. A large-scale randomized study is needed to further validate their use.

## References

[1] de Mos M., de Bruijn A. G. J., Huygen F. J. P. M., Dieleman J. P., Stricker B. H. C., Sturkenboom M. C. J. M. (2007). The incidence of complex regional pain syndrome: a population-based study. *Pain*.

[2] Sandroni P., Benrud-Larson L. M., McClelland R. L., Low P. A. (2003). Complex regional pain syndrome type I: incidence and prevalence in Olmsted county, a population-based study. *Pain*.

[3] Harden R. N., Bruehl S., Perez R. S. G. M. (2010). Validation of proposed diagnostic criteria (the ‘Budapest Criteria’) for complex regional pain syndrome. *Pain*.

[4] Harden R. N., Oaklander A. L., Burton A. W. (2013). Complex regional pain syndrome: practical diagnostic and treatment guidelines, 4th edition. *Pain Medicine*.

[5] O'Connell N. E., Wand B. M., McAuley J., Marston L., Moseley G. L. (2013). Interventions for treating pain and disability in adults with complex regional pain syndrome. *The Cochrane Database of Systematic Reviews*.

[6] Fischer S. G. L., Zuurmond W. W. A., Birklein F., Loer S. A., Perez R. S. G. M. (2010). Anti-inflammatory treatment of complex regional pain syndrome. *Pain*.

[7] Kingery W. S. (1997). A critical review of controlled clinical trials for peripheral neuropathic pain and complex regional pain syndromes. *Pain*.

[8] Bianchi C., Rossi S., Turi S., Brambilla A., Felisari G., Mascheri D. (2006). Long-term functional outcome measures in corticosteroid-treated complex regional pain syndrome. *Europa Medicophysica*.

[9] Atalay N. S., Ercidogan O., Akkaya N., Sahin F. (2014). Prednisolone in complex regional pain syndrome. *Pain Physician*.

[10] Moseley G. L., Herbert R. D., Parsons T., Lucas S., Van Hilten J. J., Marinus J. (2014). Intense pain soon after wrist fracture strongly predicts who will develop complex regional pain syndrome: prospective cohort study. *Journal of Pain*.

[11] Dijkstra P. U., Groothoff J. W., Ten Duis H. J., Geertzen J. H. B. (2003). Incidence of complex regional pain syndrome type I after fractures of the distal radius. *European Journal of Pain*.

[12] Jellad A., Salah S., Ben Salah Frih Z. (2014). Complex regional pain syndrome type I: incidence and risk factors in patients with fracture of the distal radius. *Archives of Physical Medicine and Rehabilitation*.

[13] Bruehl S. (2015). Complex regional pain syndrome. *The British Medical Journal*.

[14] Tepperman P. S., Greyson N. D., Hilbert L., Jimenez J., Williams J. I. (1984). Reflex sympathetic dystrophy in hemiplegia. *Archives of Physical Medicine and Rehabilitation*.

[15] Birklein F., Schlereth T. (2015). Complex regional pain syndrome—significant progress in understanding. *PAIN*.

[16] Parkitny L., McAuley J., Di Pietro F. (2013). Inflammation in complex regional pain syndrome: a systematic review and meta-analysis. *Neurology*.

[17] Dubuis E., Thompson V., Leite M. I. (2014). Longstanding complex regional pain syndrome is associated with activating autoantibodies against alpha-1a adrenoceptors. *Pain*.

[18] Christensen K., Jensen E. M., Noer I. (1982). The reflex dystrophy syndrome response to treatment with systemic corticosteroids. *Acta Chirurgica Scandinavica*.

[19] Kalita J., Vajpayee A., Misra U. K. (2006). Comparison of prednisolone with piroxicam in complex regional pain syndrome following stroke: a randomized controlled trial. *QJM*.

[20] Braus D. F., Krauss J. K., Strobel J. (1994). The shoulder-hand syndrome after stroke: a prospective clinical trial. *Annals of Neurology*.

[21] Kozin F., Ryan L. M., Carerra G. F., Soin J. S., Wortmann R. L. (1981). The reflex sympathetic dystrophy syndrome (RSDS). III. Scintigraphic studies, further evidence for the therapeutic efficacy of systemic corticosteroids, and proposed diagnostic criteria. *The American Journal of Medicine*.

[22] Kwon H. W., Paeng J. C., Nahm F. S. (2011). Diagnostic performance of three-phase bone scan for complex regional pain syndrome type 1 with optimally modified image criteria. *Nuclear Medicine and Molecular Imaging*.

[23] Wüppenhorst N., Maier C., Frettlöh J., Pennekamp W., Nicolas V. (2010). Sensitivity and specificity of 3-phase bone scintigraphy in the diagnosis of complex regional pain syndrome of the upper extremity. *Clinical Journal of Pain*.

